# Central Pain Processing in Chronic Chemotherapy-Induced Peripheral Neuropathy: A Functional Magnetic Resonance Imaging Study

**DOI:** 10.1371/journal.pone.0096474

**Published:** 2014-05-12

**Authors:** Elaine G. Boland, Dinesh Selvarajah, Mike Hunter, Yousef Ezaydi, Solomon Tesfaye, Sam H. Ahmedzai, John A. Snowden, Iain D. Wilkinson

**Affiliations:** 1 Academic Unit of Radiology, Faculty of Medicine, Dentistry & Health, University of Sheffield, Sheffield, United Kingdom; 2 Academic Unit of Supportive Care, Department of Oncology, University of Sheffield, Sheffield, United Kingdom; 3 Department of Diabetes, Sheffield Teaching Hospitals NHS Foundation Trust, Sheffield, United Kingdom; 4 Academic Clinical Psychiatry, Department of Neuroscience, University of Sheffield, Sheffield, United Kingdom; 5 Department of Haematology, Sheffield Teaching Hospitals NHS Foundation Trust, Sheffield, United Kingdom; University Medical Center Goettingen, Germany

## Abstract

Life expectancy in multiple myeloma has significantly increased. However, a high incidence of chemotherapy induced peripheral neuropathy (CIPN) can negatively influence quality of life during this period. This study applied functional magnetic resonance imaging (fMRI) to compare areas associated with central pain processing in patients with multiple myeloma who had chemotherapy induced peripheral neuropathy (MM-CIPN) with those from healthy volunteers (HV). Twenty-four participants (n = 12 MM-CIPN, n = 12 HV) underwent Blood Oxygen Level-Dependent (BOLD) fMRI at 3T whilst noxious heat-pain stimuli were applied to the foot and then thigh. Patients with MM-CIPN demonstrated greater activation during painful stimulation in the precuneus compared to HV (p = 0.014, FWE-corrected). Patients with MM-CIPN exhibited hypo-activation of the right superior frontal gyrus compared to HV (p = 0.031, FWE-corrected). Significant positive correlation existed between the total neuropathy score (reduced version) and activation in the frontal operculum (close to insular cortex) during foot stimulation in patients with MM-CIPN (p = 0.03, FWE-corrected; adjusted R^2^ = 0.87). Painful stimuli delivered to MM-CIPN patients evoke differential activation of distinct cortical regions, reflecting a unique pattern of central pain processing compared with healthy volunteers. This characteristic activation pattern associated with pain furthers the understanding of the pathophysiology of painful chemotherapy induced peripheral neuropathy. Functional MRI provides a tool for monitoring cerebral changes during anti-cancer and analgesic treatment.

## Introduction

Modern cancer treatments may be associated with chemotherapy-induced peripheral neuropathy (CIPN), a potentially devastating side effect. Multiple myeloma (MM) is a haematological malignancy that has a particularly high incidence of CIPN following the administration of thalidomide, bortezomib or vincristine [1], [2]. Neuropathy due to chemotherapy in myeloma may be painful and disabling. Moreover CIPN development may necessitate chemotherapy dose reduction or cessation [3], [4]. Although some resolution of CIPN is possible, there is increasing recognition of the progressive chronicity of painful neuropathy in many patients. In addition, this frequently leads to the need for long-term administration of analgesic drugs with their attendant side effect profiles. Even with current analgesics, therapeutic response is often very poor [5]. Moreover, early diagnosis and objective assessment of CIPN and its symptomatic treatments are unsatisfactory. Overall, CIPN can result in reduced quality of life and impact on survival. This runs counter to the recent achievements linked to improved life expectancy from modern anti-cancer drugs [6].

Chemotherapy-induced peripheral neuropathy is thought to be caused by drug-induced damage to components of the peripheral nervous system (PNS) [7]. Neuropathy associated with thalidomide is thought to arise from Wallerian degeneration of the peripheral nerve fibres contributing to the development of pain via production of cytokines such as tumour necrosis factor-α and nerve growth factors [8]. Bortezomib-induced neuropathy is associated with deficits in Aβ, Aδ and C primary afferent fibres. There could potentially be a dysregulation of the neurotrophic factors since bortezomib inhibits the activation of the transcription factor, nuclear factor-kappa B and blocks the transcription of the trophic nerve growth factor [2]. Vincristine acts by binding on intracellular tubulin, which interferes with axonal transport. It is thought that it induces alterations in the cellular microtubule structure in the PNS and this might be a mechanistic link to neuropathic development [9], [10]. Neuropathic symptoms are usually more common and severe in the lower extremities than in the upper extremities as the longest nerves are thought to be the first affected. Sensory changes affecting the toes, finally progress proximally to the ankles in a glove and stocking distribution [11]. Structural damage to the PNS results in abnormal somatosensory processing in the central and peripheral nervous systems [12]. Most of the current ideas regarding the pathophysiology of neuropathic pain originated from experimental work in animal models indicating peripheral and spinal cord reorganization of nociceptive pathways [13]. Animal models of neuropathic injuries that result in persistent pain suggest that peripheral and spinal cord impulses transmitting nociceptive signals towards the cortex undergo remarkable reorganization [14], [15]. Although peripheral abnormalities are prominent, the central nervous system (CNS) appears to show concomitant involvement. Abnormalities in pain processing as shown on neuroimaging rather than just damage or inflammation to the peripheral nerves, seem to play an important part in chronic painful conditions such as trigeminal neuralgia, post-herpetic neuralgia, chronic low back pain and diabetic neuropathy [16]–[20]. Areas of brain activation that have been reported in chronic pain include the frontal lobe, insular cortex, somatosensory cortex, thalamus, periaquedactal grey and precuneus [21]–[23].

Our understanding of the pathological mechanisms underlying CIPN and identification of possible future treatment target areas for this debilitating complication may be significantly enhanced by the application of functional magnetic resonance imaging (fMRI). To our knowledge, to date there have been no reported fMRI studies in patients with multiple myeloma who have developed treatment emergent CIPN, that have attempted to document pain processing within the brain.

The aim of this work was to assess any alterations to the brain's cortical and sub-cortical pain matrix in MM-CIPN due to factors including: chemotherapy, chronicity of painful neuropathy and psychological adaptation. In light of previous pain-related functional studies, we hypothesised that regions such as the frontal cortex, insula, and postcentral gyrus, known to be involved in cognition and emotion processing and sensory perception, would be modulated by the presence of post-chemotherapeutic chronic neuropathy. To test this hypothesis, we used Blood Oxygenation Level Dependent (BOLD) fMRI to study central pain processing during noxious thermal stimulation in patients with MM who had already developed CIPN and to compare these findings to those from healthy volunteers (HV).

## Material and Methods

### Ethics statement

The study was granted ethical approval by the Sheffield Research Ethics Committee (REC no: 08/H1308/276) and the participants' written informed consent was obtained in accordance with the Declaration of Helsinki.

### Subjects

The study sample comprised MM-CIPN patients and healthy volunteers. Myeloma patients had been treated with at least one of the anti-myeloma therapies commonly associated with CIPN (i.e; thalidomide, bortezomib or vincristine) and had experienced neuropathic pain of at least six months duration. Exclusion criteria included history of any major neurological or psychiatric disorder, contraindications to MRI, claustrophobia, left hand dominance, and neuropathy caused by other medical conditions. In addition, MM-CIPN patients who could not discontinue their tricyclics, noradrenaline reuptake inhibitors and/or calcium channel blocker analgesic medication for at least 48 hours prior to scanning were excluded. Patients who were on a long term, stable dose of opioids were included. Subjects did not receive any financial incentive for their participation. For each subject, demographic data collection, neuropathy assessment, chronic pain assessment and MR imaging were all undertaken on the same day.

### Neuropathy assessment

All subjects underwent detailed clinical and neurophysiological assessments to diagnose and quantify the presence of neuropathy. This included a detailed clinical history and examination, quantitative sensory testing and peripheral nerve conduction assessment. Sensory testing using Computer Assisted Sensory Evaluation IV equipment comprised vibration perception, cooling and heating detection threshold of the right foot (CASE IV) (W.R. Electronics, Stillwater, MN, USA). Peripheral nerve conduction studies were performed at a stable skin temperature of 31°C (Medelec electrophysiological system, Synergy Oxford Instruments, Oxford, U.K.). A total neuropathy score reduced version (TNS-reduced version) was used, as has previously been used to stage neuropathy in MM patients [24]. This score evaluates motor and sensory symptoms and signs, quantitates pinprick and vibration perception threshold, and includes neurophysiological examination of one motor and one sensory nerve in the leg. The TNS-reduced version composite score ranges from 0 to 32; and the presence of neuropathy was defined as having a TNS-reduced version score >2.

### Chronic pain assessment

To cover the psychosocial consequences of the pain experienced by the MM-CIPN patients, additional assessment tools were used. The chronic pain acceptance questionnaire (CPAQ) provides information on how chronic pain affects patients and is rated on a 0 (never true) to 6 (always true) scale [25]. The CPAQ total score ranges from 0 to 120, with a higher score indicating higher acceptance of pain. It shows the level of acceptance of chronic pain and focuses on participation in activities, the pursuit of personally relevant goals and the relative absence of attempts to control or avoid pain.

The pain catastrophizing scale (PCS) is a 13-item self-report test whose items are rated on a 5-point Likert-type scale. Ratings range from 0 (not at all) to 4 (always) representing the degree to which the subject experienced each of 13 thoughts or feelings when in pain [26]. The PCS total scores range from 0–52. The items fall within three different categories to assess negative thinking styles related to pain: rumination, magnification, and helplessness.

### Heat-pain stimulation protocol

All heat-pain stimulation was applied to the right hand side of the body for each participant. The experimental thermal stimulus was delivered using a computer-controlled, MR-compatible, contact heat evoked potential device (CHEPS), which rapidly delivers heat with controllable temperatures (Medoc Pathway System, Medoc Ltd, Ramat Yishai, Israel). The foot was chosen as the primary stimulation area as this is the anatomical location where patients with CIPN develop pain-related symptomatology. The thigh was the secondary stimulation site and it was chosen as a potential control site, not being associated with neuropathic pain.

Prior to each subject entering the MR scanner, a simple heat pain-rating test was performed to determine the temperature threshold necessary to elicit a score of at least 7 on an 11-point numeric rating scale (with reference to a 0–10 point numeric rating scale; where 0 represented no pain and 10 represented the most extreme pain). This was performed separately for stimulation at both foot and thigh. The pain rating protocol started with a temperature application of 44.9°C at the foot and 42.9°C at the thigh. At these and subsequent temperature iterations, each subject reported their subjective pain status. The temperature was increased or decreased by 0.5–2°C until a pain score of 7/10 was identified. The maximum temperature at either site was set at 47.9°C. Thermal stimulation has been consistently used in other fMRI studies and has been shown to be an effective way to describe brain activity maps in response to thermal pain [27].

Each fMRI run comprised the presentation of 7 blocks of heat-pain stimuli (duration 30 seconds), at the pre-determined 7/10 pain-inducing temperature, interspersed with 7 blocks at a baseline temperature of 32°C (each baseline block duration was pseudo-randomised to 55, 60 or 65 seconds). Timing of the applied temperature protocol was controlled by the pre-programmed thermode-device computer. At the end of the imaging session, all participants rated their overall pain when the heat-pain stimulus was delivered to the foot and to the thigh. This was done by asking subjects to verbally rate the intensity of the pain on a numerical scale from zero (no pain) to ten (maximum imaginable pain).

### Scanning protocol and fMRI acquisition

A fixed scanning protocol was used for every subject. Magnetic Resonance data were acquired at 3 Tesla (Achieva 3.0T, Philips Healthcare, Best, Holland). The scanning protocol included acquisition of standard T1-weighted, 3-dimensional and T2-weighted, 2-dimensional anatomical images prior to two fMRI runs. The fMRI datasets were acquired using a single-shot, T2*-weighted, gradient-recalled, echo planar imaging (EPI) sequence (Time to echo (TE)  = 35 ms; Time to repeat (TR)  = 3000 ms; SENSE encoding factor  = 1.5). For each dataset, 35 contiguous transaxial slices of 4 mm thickness, with an in-plane resolution of 1.8 mm×1.8 mm were acquired at each imaging time-point or temporal dynamic (phase encoding in the anterior-posterior direction). Synchronicity was maintained between the scanner fMRI acquisition and the thermode-device PC control.

### fMRI analysis

Imaging data analysis was carried out using statistical parametric mapping software (SPM5: www.fil.ion.ucl.ac.uk/spm). Blood oxygen level dependent response was modelled using a box-car waveform convolved with a canonical haemodynamic response function. Following spatial pre-processing, which included realignment, spatial normalisation and spatial smoothing, images were analysed using the General Linear Model. First-level functional images were produced comparing the BOLD response under the heat pain condition with the BOLD signal obtained at the baseline temperature. These contrast estimate maps were produced for every functional run at each of the two anatomical stimulation sites (foot and thigh) for each subject. Therefore, the first-level analysis yielded 2 contrast images per subject, comparing BOLD response under the heat-pain condition with baseline at (i) foot and (ii) thigh stimulation sites.

The resultant first-level contrast images were combined at the group level in a flexible factorial model with factors of subject, group (MM-CIPN and healthy volunteers) and site (foot and thigh). This appropriately modelled the independence of observations between subjects and groups, and the inequality of variance across all factors.

Within-group images of all pain (foot and thigh) response versus baseline were produced for each of the MM-CIPN and healthy volunteer groups. The statistical threshold for reporting within-group pain contrast activation was p<0.05, family-wise error (FWE) corrected across the whole spatial brain volume.

A functional anatomical mask of voxels activated in response to all heat-pain stimuli (compared with baseline) in both groups of subjects (MM-CIPN and healthy volunteers) was created at a voxel-level statistical threshold p<0.001, uncorrected. This masking approach was used in order to reflect any variance in functional-anatomical location resulting from disease-specific pathogenesis. The mask was used as a volume-of-interest for correction for multiple comparisons in subsequent between-group contrasts.

The statistical threshold for reporting between-group differences in pain-evoked activation was p<0.05 FWE corrected (voxel-level) or p<0.05 corrected for extent of activation (cluster level) in either the whole brain volume or functionally defined region-of-interest (above). All activation results were displayed in the anatomical space as defined by the Montreal Neurological Institute (MNI) with stereotactic co-ordinates converted to the standard space of Talairach and Tournoux for the purposes of neuroanatomical labelling [28], [29].

### Behavioural data statistical analysis

Quantitative data were analysed using PASW Statistics version 18 [30]. Because of the asymmetric distribution of the cohort, a non-parametric (Mann-Whitney U test) was used for group comparisons. A linear regression analysis of fMRI signal response to heat-pain stimulation at the foot (i.e., neuropathic site) within the MM-CIPN patient group with TNS-reduced version and with the CPAQ and the PCS questionnaire scores was performed.

## Results

### Baseline characteristics

Twenty-four subjects were recruited (table 1): twelve MM-CIPN patients (8 males) and 12 healthy volunteers (6 males). The median age of the myeloma group was 63 years whilst that of the healthy volunteers was 53 years. Myeloma patients had the disease for a median [inter-quartile range (IQR)] of 4 [2–7.7] years and had neuropathic pain for a median of 2 [0.9–3.2] years. The anti-myeloma therapy that these patients received, which are known to cause peripheral neuropathy, varied between patients: n = 5 (42%) vincristine, n = 9 (75%) thalidomide and n = 8 (67%) bortezomib. All participants completed the study and none were excluded.

**Table 1:** Baseline characteristics (median [IQR]) of the subjects recruited.

	Multiple Myeloma patients	Healthy Volunteers	p-value
**Group Size**	12	12	0
**Age (Years)**	63 [56–67]	53 [35–58]	0.04
**Sex**	8 Males;4 Females	6 Males;6 Females	0.42
**Duration of MM (Years)**	4.1 [2.0–7.7]	Not applicable	0
**Duration of neuropathic pain (Years)**	2 [0.9–3.2]	Not applicable	0

### Neurological assessment and pain stimulation

Neurophysiological tests showed abnormality in each MM-CIPN subject indicative of peripheral neuropathy in the feet. The median [IQR] of the TNS-reduced version for MM-CIPN patients was 14 [11]–[17]. A length-dependent sensory axonal, large fibre neuropathy was evident from the nerve conduction studies. This showed smaller amplitudes and longer latencies and also impaired vibration and cooling thresholds on quantitative sensory testing.

The subject-reported rating for pain intensity was recorded before and after MRI scanning as shown in table 2. There were no significant group differences (p>0.05) in median temperatures applied to either the foot or thigh. There were no significant differences (p>0.05) between pre-scanning and post-scanning pain intensity rating in either healthy volunteers or MM-CIPN groups.

**Table 2:** Group median [IQR] temperature for pain stimulation and pain rating.

	Healthy Volunteers	MM patients	p-value
**Foot stimulation**			
**Temperature (°C)**	46.7 [45.5–47.8]	47.2 [46.6–47.9]	0.23
**Pre-scan pain rating (0–10)**	7.5 [6.6–8.0]	7.0 [7.0–8.0]	0.74
**Post-scan pain rating (0–10)**	7.7 [6.6–8.7]	7.7 [7.0–8.0]	0.95
**Thigh stimulation**			
**Temperature (°C)**	45.9 [45.6–47.9]	46.7 [45.5–47.9]	0.88
**Pre-scan pain rating (0–10)**	7.7 [5.9–8.0]	8.0 [7.6–8.0]	0.28
**Post-scan pain rating (0–10)**	8.0 [6.2–8.9]	8.0 [8.0–9.0]	0.29

### Chronic pain assessment

The total median CPAQ score in the MM group was 75. The median sum score for activities engagement was 45 and that of pain willingness was 31.

The total median catastrophisation score on the PCS was 11 (range 0–52) with the median scores (ranges) for magnification, rumination and helplessness being 2.5 (0–12), 5 (0–16) and 4.5 (0–24) respectively.

### Brain activation in response to heat-pain stimulation in both healthy volunteers and MM-CIPN patients

#### Individual group analysis

Separate statistical parametric activation maps were constructed for the MM-CIPN group and the healthy volunteers group. Compared with baseline, heat-pain stimulation evoked a BOLD response or ‘activation’ in both healthy volunteers and patients with MM- CIPN (Table 3).

**Table 3:** Anatomical regions showing areas where statistical significant BOLD fMRI response to heat-pain stimuli were greater than baseline temperature for Healthy Volunteers and myeloma patients with CIPN.

Healthy volunteers: Pain>Baseline					
Region	BA	Talairach co-ordinates	Peak t	p (FWE-corr)	Voxels*
R anterior cingulate	32	4 24 23	7.47	<0.001	1643
L superior temporal gyrus	38	−50 13 −8	7.19	<0.001	1491
L posterior cerebellar lobe	-	−30 −67 −20	6.56	0.002	2917
R inferior frontal gyrus	47	44 20 −9	6.52	0.002	1151
R supramarginal gyrus	40	64 −41 34	6.36	0.004	687
L superior temporal gyrus	42	−60 −30 18	6.13	0.007	709
L superior frontal gyrus	10	−32 48 20	5.80	0.018	469
R middle frontal gyrus	46	42 44 24	5.73	0.02	390
R superior frontal gyrus	8	6 39 48	5.68	0.025	216
L postcentral gyrus	7	−20 −41 68	5.66	0.026	212

CIPN-myeloma patients: Pain>Baseline					
Region	BA	Talairach co-ordinates	Peak t	p (FWE-corr)	Voxels*
R superior temporal gyrus	22	56 4 0	8.10	<0.001	1356
R supramarginal gyrus	40	62 −41 35	7.65	<0.001	735
L middle frontal gyrus	46	−34 40 22	6.25	0.005	544
L area opercularis	44	−54 2 12	6.07	0.008	2228
L posterior cerebellar lobe	-	−34 −58 −24	6.00	0.01	2535

*Voxels  =  number of voxels exceeding threshold p<0.001, uncorrected

#### Comparison between groups

**Figure 1:**
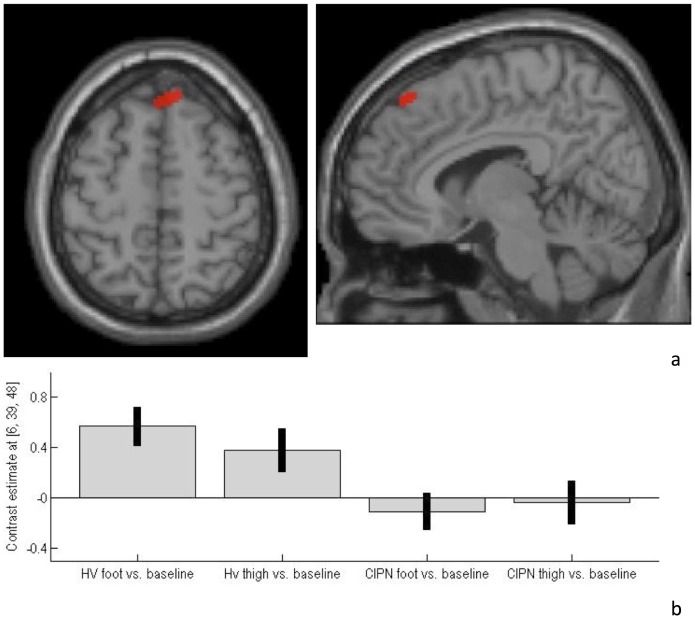
CIPN-myeloma patients demonstrated a) hypo-activation of superior frontal gyrus during heat-pain stimulation, compared with healthy volunteers. Functional imaging data are shown overlaid on both axial (z = 48 mm) and sagittal (x = 6 mm) slices through a canonical single-subject T1-weighted image. For display purposes, the statistical threshold is p<0.001, uncorrected, at the voxel-level. **b) Contrast estimates and 90% CI at co-ordinate 6, 39, 48 for both healthy volunteer and CIPN-myeloma patient groups.**

Patients with MM-CIPN demonstrated significantly less activation in the right superior frontal gyrus, close to the midline, than the healthy volunteer group (Figure 1a; Brodmann area 8; Talairach co-ordinates: 6, 39, 48; peak t = 4.87; p = 0.03, FWE-corrected within the volume-of-interest; 97 voxels exceeded height threshold p<0.001, uncorrected). Contrast estimates for pain stimulation at this focus, according to group and stimulation site, are presented (Figure 1b).

**Figure 2:**
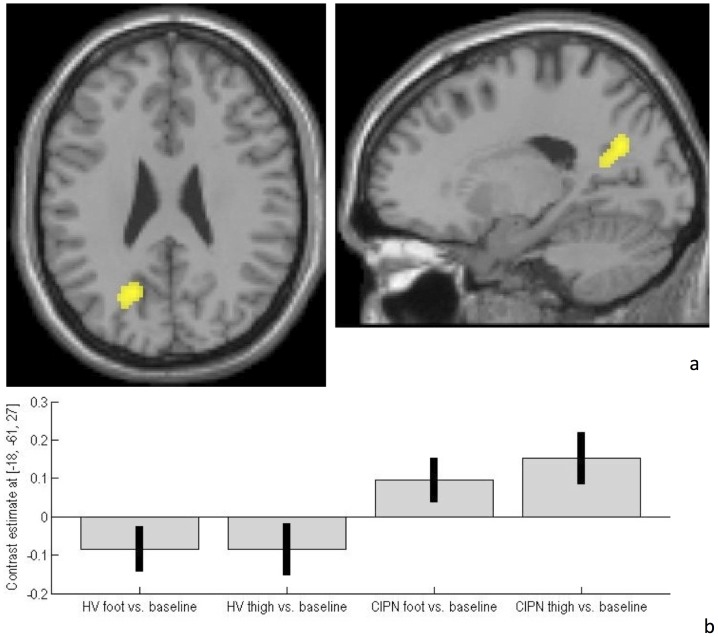
CIPN-myeloma patients demonstrated a0 hyper-activation of precuneus during heat-pain stimulation, compared with healthy volunteers. Functional imaging data are shown overlaid on both axial (z = 27 mm) and sagittal (x = −18 mm) slices through a canonical single-subject T1-weighted image. For display purposes, the statistical threshold is p<0.001, uncorrected, at the voxel-level. **b) Contrast estimates and 90% CI at co-ordinate −18, −61, 27 for both healthy volunteer and CIPN-myeloma patient groups.**

Compared with healthy volunteers, MM-CIPN patients demonstrated significantly greater activation in left precuneus (Figure 2a; Brodmann area 31; Talairach co-ordinates: −18, −61, 27; peak t = 4.82; 244 voxels exceeded height threshold p<0.001, uncorrected; p = 0.01, corrected for extent of activation). This can be visualised in the plots for the contrast estimates for pain stimulation at this focus, according to group and stimulation site (Figure 2b).

### Correlation of brain activation with the total neuropathy score, reduced version

**Figure 3:**
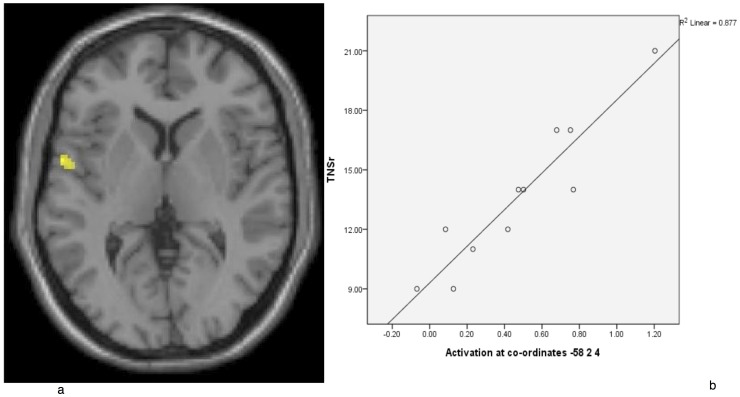
a: CIPN-myeloma patients demonstrated activation in the operculo-insular cortex. This activation was during painful heat stimulation, the degree of which correlated with TNS-reduced version. For display purposes, the statistical threshold is p<0.001, uncorrected, at the voxel-level. **b: Linear correlation between the TNS-reduced version and BOLD response within the operculo-insular cortex.**

A linear regression analysis of fMRI signal response to heat-pain stimulation at the foot (i.e., neuropathic site) within the MM-CIPN patient group indicated significant correlation of BOLD response with TNS-reduced version in the left operculo-insular cortex (Figure 3a, 3b, Talairach co-ordinates: −58, 2 4; peak t = 8.02; p = 0.03 FWE-corrected in the volume-of-interest for MM-CIPN foot activation).

No significant correlations were identified in the MM-CIPN patient group between BOLD response and CPAQ and/or the PCS questionnaire scores.

## Discussion

This is the first report of a fMRI study exploring central pain processing in myeloma patients with chemotherapy-induced peripheral neuropathy. In this initial study, a thermal stimulus was applied to probe the subjects' BOLD-fMRI response to acute heat-pain. This type of stimulus was chosen since ‘burning pain sensations’ often feature strongly in CIPN clinical symptomatology. Our findings in patients who had this complex pathology mirror and underpin the central changes in other neuropathic pain conditions reported in the literature. There was significant activation of the precuneus in the MM-CIPN group during heat-pain stimulation whilst there was significantly less response observed in this area in healthy volunteers. The precuneus, which lies on the posteromedial aspect of the parietal lobe, is thought to play a role in conscious pain perception and is involved in a broad range of higher order cognitive functions. Activation of the posterior precuneus exhibits the strongest correlation with successful retrieval of remembered episodes [31]. In MM-CIPN patients, the enhanced precuneus response to painful stimulation could imply that pain stimulates episodic pain memory retrieval. This is consistent with the literature in normal individuals [32], . It has also been shown in patients with other chronic neuropathic conditions [23], [34]–[36].

In the MM-CIPN patients, there was a significant positive correlation between increasing neuropathy score (TNS-reduced version) and BOLD response in the operculo-insular cortex. This region includes parts of the insula deep inside the lateral sulcus. It also includes parts of the frontal and parietal lobes that cover the insula, called the opercula. The operculum is known to play a part in the cortical processing of painful stimuli [37]. There is evidence to show that the intensity of activation of the operculo-insular cortex correlates with perceived pain intensity in the human brain [38].

Interestingly, in our cohort of MM-CIPN patients, no functional anatomical response was identified in the superior frontal gyrus. This could result from adaptation due to the chronic presence of painful neuropathy of at least two years duration. Patients reported high scores in the chronic pain acceptance questionnaire and low scores in the pain catastrophizing questionnaire, which supports the hypothesis that reduction in BOLD fMRI response to acute heat-pain stimulation follows from long-term neuropathy-related pathophysiology. Previous studies have highlighted deactivation of the prefrontal cortex in response to pain in cluster headaches [39]. Repetitive transcranial magnetic stimulation has also been linked to deactivation in capsaicin-induced pain [40]. Activation in the right superior frontal gyrus has been previously reported in studies applying painful stimuli to healthy volunteers and activation in this area has been attributed to both the subjective experience of pain and other general unpleasant experiences [41].

We identified regions that are known components of the brain's pain matrix, during painful stimulation in both healthy volunteers and MM-CIPN patients. These functional areas are comparable to those found in other disease groups [23], [42]–[44].

There are numerous factors that may influence the overall *in-vivo* fMRI experiment [45]. In the current ‘box-car’ design, the BOLD effect is based on changes in MR signal between functional states, the source of which is assumed to be dominated by the net haemodynamic change linked to localised differences in synaptic and neuronal firing. It is not a direct measure of neuronal activity; rather it is an indirect physiological response. The vascular coupling is currently not fully understood, particularly in the context of pathophysiology. One potential limitation of this study was that the median age of the MM-CIPN group was greater than that of the healthy volunteers (albeit similar, approximately 10 years). The reported influences of age on BOLD fMRI response for non-pain-specific studies appear complex including overall intracranial increases, increases in frontal regions and decreases in the anterior regions, all with increasing age (a proposed posterior-anterior shift with age reported between 22- year and 69- year old normal volunteer groups) [46]. It must also be noted that chemotherapeutic agents themselves may alter vascular function and thus results should be interpreted in the light of these potential modulatory factors. However, as effects caused by alterations in cardiac output are likely to be systemic in nature and the group age difference in this study being comparatively small, it is assumed that potential effects between different intra-cranial functional areas are secondary to the proposed neuropathic effects. The significant correlation found with increasing neuropathic severity supports this hypothesis. Additionally, in terms of group response to pain-provoking stimulation, there were no significant differences between median temperatures that were delivered and their resultant pain ratings to either of the groups.

This was a cross-sectional screening study, with myeloma patients having already developed neuropathy in the context of receiving different anti-myeloma therapies. Some therapeutic regimens included more than one possible CIPN-inducing agent. Since no other known major risk factors for peripheral neuropathy were identified, such as diabetes, human immunodeficiency virus infection, chronic alcoholism, amyloidosis or renal failure, it would seem that a chemo-therapeutic response was the major overall causal factor linked to differences in both peripheral and central nervous systems. In addition, although peripheral neuropathy is seen at presentation in a small minority of patients with untreated MM, in our study, neuropathic pain started only after having received one of the anti-myeloma therapies described above. Most drugs used as an anti-myeloma treatment cannot permeate the blood–brain barrier [47]. The blood-nerve barrier protecting the peripheral system does however appear to be sensitive to the aforementioned agents. The PNS is thus more readily affected by circulating drug neurotoxicity than CNS structures [48]. In the light of this, the observed cerebral differences in BOLD response to heat stimulation may result from changes in sensory input to the brain or feedback circuitry via the spinal cord/brainstem. Longitudinal data from patients with myeloma embarking on chemotherapy would help to identify that CNS alterations do not occur prior to peripheral changes and thus the primary effect-site being the PNS. Such data would also help elucidate whether CNS and PNS changes occur concurrently or whether alterations in cerebral response follow a time lag suggestive of, for example, brain function accustomisation. This preliminary study compared a MM-CIPN cohort to findings in a healthy control group. Further data obtained from disease-control groups, consisting of patients with MM who have not developed symptoms or signs associated with peripheral neuropathy and those in the acute phase who have recently developed neuropathy, will add to our understanding of neuropathic pathogenesis. Patients receiving long-term opioid medication were not excluded and their medication was not withdrawn, to facilitate involvement and to sample the patients in their clinically relevant, chronic CIPN-state. It should be noted that future studies might reveal the potentially interesting modulatory effects of this psychoactive agent.

In conclusion, our results indicate that fMRI is capable of providing novel information with respect to the processing of pain within the CNS in CIPN. The results show that painful stimuli delivered to neuropathy-affected and symptom-free sites in our patients with CIPN evoked differential activation of distinct cortical regions. From this, we infer that the nociceptive system may undergo plastic changes, which appear to reflect abnormal central pain processing. Larger patient cohorts are needed to ensure that the current findings represent those of CIPN in general. Further identification and understanding of pathology-specific CNS involvement may provide an objective, in-vivo-based measure of pain experience and perception in patients with CIPN. This may aid in diagnosis and may also provide adjunctive information with which to steer targeting of novel pain-relieving therapies.
